# Mogroside V Alleviates Lipopolysaccharide-Induced Neuroinflammation via Inhibition of TLR4-MyD88 and Activation of AKT/AMPK-Nrf2 Signaling Pathway

**DOI:** 10.1155/2021/5521519

**Published:** 2021-04-30

**Authors:** Yuanyuan Liu, Boxi Zhang, Jiahe Liu, Chunyu Qiao, Nianyu Xue, Hongming Lv, Shize Li

**Affiliations:** College of Animal Science and Veterinary Medicine, Heilongjiang Bayi Agricultural University, Daqing 163319, Heilongjiang Bayi, China

## Abstract

As innate immune effector cells in the central nervous system (CNS), microglia not only are essential for the normal development of nervous system but also act on different neurological diseases, including Alzheimer's disease (AD), Huntington's disease (HD), and other neuroinflammatory diseases. Mogroside V (Mog), a natural plant active ingredient and isolated form of *Momordica grosvenori*, has been shown to possess anti-inflammatory action, but few studies were carried out to investigate the effects of Mog on neuroinflammation. This study aimed to investigate the role of Mog in lipopolysaccharide- (LPS-) induced neuroinflammation and neuronal damage, revealing the underlying mechanisms. Our data indicated that Mog significantly inhibited the LPS-induced production of proinflammatory factors, such as tumor necrosis factor-*α* (TNF-*α*), interleukin-1*β* (IL-1*β*), IL-18, IL-6, cyclooxygenase-2 (COX-2), inducible nitric oxide synthase (iNOS), and high mobility group box 1 (HMGB1) in BV-2 cells. We found that Mog also suppressed toll-like receptor 4 (TLR4), myeloid differentiation factor 88 (MyD88), the phosphorylation of mitogen-activated protein kinases (MAPKs), adenosine 5′-monophosphate- (AMP-) activated protein kinase (AMPK), nuclear factor kappa-B (NF-*κ*B), and protein kinase B (AKT). Moreover, Mog also enhanced the expression of *γ*-glutamyl cysteine synthetase catalytic subunit (GCLC), modifier subunit (GCLM), heme oxygenase-1 (HO-1), and quinine oxidoreductase 1 (NQO1) proteins, mostly depending on the nuclear translation of nuclear factor erythroid-2 related factor 2 (Nrf2). In contrast, pretreatment with inhibitors of AKT can suppress the phosphorylation of AMPK, Nrf2, and its downstream proteins expression. In summary, Mog might play a protective role against LPS-induced neurotoxicity by inhibiting the TLR4-MyD88 and activation of AMPK/AKT-Nrf2 signaling pathway.

## 1. Introduction

Neuroinflammation is recognized as a vital pathological process for various neurodegenerative diseases [1]. Under normal conditions, as brain resident macrophages, microglia can protect nervous system from a series of injuries by removing protein aggregates and phagocytosing debris [2]. However, when microglia are subjected to stress and noxious stimuli, they are activated, thus releasing inflammatory mediator and proinflammatory factor and further resulting in neurodegenerative diseases, including AD, Parkinson's disease (PD), spinocerebellar ataxias (SCA), and chronic pain pathologies [3–6]. Therefore, alleviated neuroinflammation will probably become an effective treatment method for neurodegenerative diseases.

LPS is a composition in the outer wall of Gram-negative bacteria cell wall. As a ligand, it can be recognized by TLR4 [7], and it is highly expressed in microglia [8]. Via activation of TLR4, recruitment of MyD88, and activation of NF-*κ*B, LPS-activated microglia lead to the increase of various inflammatory mediator expressions, including iNOS and COX-2, and proinflammatory cytokines, such as TNF-*α*, IL-1*β*, IL-18, and IL-6 [9, 10]. In addition, TLR4 mediated downstream MAPKs, including p38 MAPK, extracellular signal-regulated protein kinase 1/2 (ERK1/2), and c-Jun amino-terminal kinase (JNK) that are increased by LPS stimulated and involved various inflammation reactions, thereby leading to neuroinflammatory aggravation [11]. Nrf2 is a transcription factor, and it is mainly mediated inflammation and oxidative stress [12]. Meanwhile, when Nrf2 suffered from the stimulation of inflammation, it enters nucleus and further causes a series of increasing downstream proteins, such as NQO1, HO-1, GCLC, and GCLM [13]. In addition, studies proved that AKT and AMPK are key energy sensors of cellular metabolism in response to inflammation and act as upstream kinases of Nrf2 and then take part in regulating anti-inflammation [14, 15].

**Figure 1:**
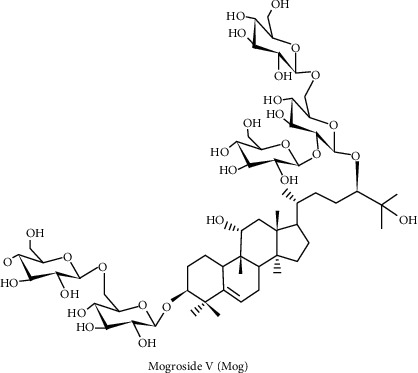
The chemical structure of Mogroside V.

Monk fruit is the fruit of the plant Luo Han Guo (LHG); this plant is a perennial vine in the Cucurbitaceae family. It bears round green fruit, and the chemical structure of the sweet ingredient in the fruit belongs to triterpenes and is named Mogroside [16]. Mogroside (Figure 1) belongs to cucurbitane-type glycosides that are extracted from fructus of *Siraitia grosvenorii*, and it grows in Guangxi province in southern China [17]. According to historical records, the fructus of *Siraitia grosvenorii* has several effects, such as profit lung, laxative, clear heat, and treatment of sore throat [18]. Moreover, according to numerous studies, Mogrosides also display multiple pharmacological effects on cancer, oxidative stress, inflammation, obesity, and diabetes [18–20]. Mog is one of the most effective components of Mogrosides. Recent researches have displayed that Mog has anti-inflammatory function in the LPS-induced mouse acute lung injury (ALI) model via the activation of NF-*κ*B signaling pathway [21]. Moreover, Mog also inhibited proinflammatory factors expression, including COX-2 and iNOS that are induced by LPS in macrophages [22]. Nie et al.'s experimental results demonstrate that Mog can alleviate the deterioration of oocyte quality during in vitro aging and possibly by upregulating SIRT1 to reduce oxidative stress [23]. However, up to now, the role of Mog in LPS-induced neuroinflammation still remains unclear.

However, whether Mog can protect against neurocyte injury is still unknown. Therefore, the aim of the present study was to explore the effects of Mog in LPS-induced microglia activation and further illustrate Mog-mediated signaling pathways of contributing to the beneficial effects and providing experimental evidence to develop new drug candidates against neuroinflammation.

## 2. Materials and Methods

### 2.1. Chemicals and Reagents

Mog (HPLC > 98%) was obtained from Chengdu Pufei De Biotech Co., Ltd. (Chengdu, China; CAS:88901-36-4). The compound was dissolved in dimethyl sulfoxide (DMSO, 0.1% final concentration in cultural medium). DMSO, LPS (*Escherichia coli* Serotype 055:B5), and LY294002 (10 *μ*M, AKT inhibitor) were purchased from Sigma Chemical Co. (St Louis, USA). 3-[4,5-dimethylthiazol-2-yl]-2,5-diphenyltetrazolium bromide (MTT) and phosphate-buffered saline (PBS) were supplied by Coolaber (Beijing, China). BCA were obtained from Beyotime Biotechnology (Shanghai, China). ECL chemiluminescence substrates were supplied from Millipore Corporation (Billerica, USA). COX-2, iNOS, TLR4, Nrf2 HMGB1, IL-1*β*, IL-18, NF-*κ*B, p-NF-*κ*B, MyD88, p-AKT, AKT, p-AMPK, AMPK, IL-6, and TNF-*α* were obtained from ABclonal Technology (Wuhan, China). Rabbit polyclonal antibodies against ERK (#4695), p-ERK (#4370), JNK (#9252), p-JNK (#4668), p38 MAPK (#8690), p-p38 (#4511), I*κ*B*α* (#4814), and p-I*κ*B*α* (#2859) were purchased from Cell Signaling Technology (Bossdun, USA). NQO1 (ab80588), HO-1 (ab68477), GCLC (ab207777), and GCLM (ab126704) were provided by Abcam (Cambridge, United Kingdom). Dulbecco's modified Eagle's medium (DMEM) and trypsin were provided by Gibco (USA). Fetal bovine serum (FBS) was from Biological Industries (Uruguay, South America).

### 2.2. Cell Culture

The BV-2 cells were purchased from Shanghai Cell Bank, Chinese Academy of Sciences. The BV-2 cell lines and SH-SY5Y neurons were cultured in DMEM supplemented with 10% FBS, 100 U/mL penicillin, and 100 *μ*g/mL streptomycin (Gibco) and incubated at 37°C in a humidified incubator containing 95% air and 5% CO_2_ atmosphere. Because FBS contains numerous compounds, such as LPS and growth factors which influence the biological characteristics of cells, we performed all experiments under serum-free conditions; and in our experiments, cells were allowed to accommodate for 24 h before any treatment.

### 2.3. Cell Viability Assay

BV-2 cells were seeded in 96-well culture plates at a density of 2.5 × 10^3^ cells/well. The cells were pretreated with serial concentrations of Mog or LPS exposure as described below. To each well, 10% MTT solution (5 mg/mL) was added and incubated at 37°C for another 3 h. Cell-free supernatants were then removed and cells resolved with 100 *μ*L/well DMSO. The optical density was measured at 570 nm on a microplate reader.

To measure the anti-inflammation potential of Mog against the LPS-induced neuronal damage in SH-SY5Y neurons, the BV-2 cells and SH-SY5Y neurons were treated with 100 *μ*L different concentrations of Mog (0, 6.25, 12.5, and 25 *μ*M) for 2 h, followed by stimulation with 1 *μ*g/mL of LPS for 18 h. Subsequently, the conditioned media from the BV-2 cells cultures were treated with SH-SY5Y neurons for 24 h. To each well, 10 % MTT solution (5 mg/mL) was added and incubated at 37°C for another 3 h. Cell-free supernatants were then removed and cells resolved with 100 *μ*L/well DMSO. The optical density was measured at 570 nm on a microplate reader.

### 2.4. Western Blot Analysis

Cells were collected and lysed in NP-40 cracking liquid with 1 mM phenylmethanesulfonyl fluoride (PMSF). The cells were placed in a 4°C refrigerator for 30 min to fully lyse. The supernatant was carefully transferred into new Eppendorf tubes after centrifuging at 12,000 rpm at 4°C for 10 min and preserved at −20°C until further use. Then protein concentration was determined by BCA protein assay kit; and promised equal amounts of protein (20 *μ*g) were separated by a 12.5% SDS-polyacrylamide gel and transferred onto a polyvinylidene difluoride (PVDF) membrane. Then the membrane was blocked with 5% skimmed milk powder solution for 2 h at room temperature. After blocking, the membrane was incubated with primary antibodies at 4°C overnight. The next day, it was washed in Tris-buffered saline with Tween 20 (TBST, 0.05%) and washed 5 times each 5 minutes. Subsequently, the membrane was incubated with HRP secondary antibody. Blots were then developed with the ECL Plus Western Blotting Detection System (Amersham Life Science, UK).

### 2.5. Statistical Analysis

Results are reported as the mean ± SEM and were statistically analyzed by means of analysis of variance (ANOVA) followed by the Student–Newman–Keuls test. All tests were performed in GraphPad Prism 5.00 (GraphPad Software, La Jolla, CA, USA). *p* ≤ 0.05 were regarded as significant.

## 3. Result

### 3.1. Effects of Mog and LPS on BV-2 Cells and SH-SY5Y Neurons Viability

**Figure 2:**
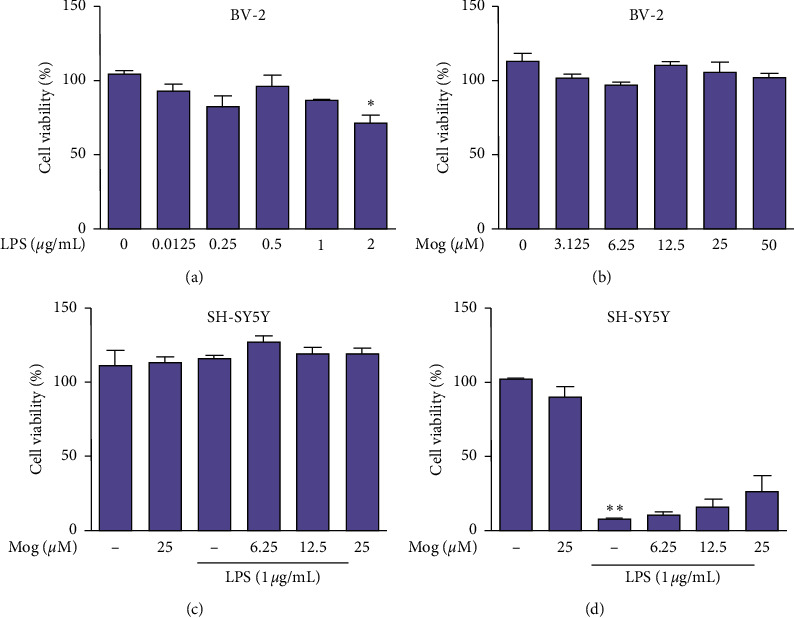
Effects of Mog and LPS on BV-2 cells and SH-SY5Y neurons viability. (a) BV-2 cells were treated with different concentrations (0, 0.125, 0.25, 0.5, 1, and 2 *μ*g/mL) of LPS for 24 h and the cell viability was determined by MTT assay. (b) BV-2 cells were treated with different concentrations (0, 3.125, 6.25, 12.5, 25, and 50 *μ*M) of Mog for 24 h and the cell viability was determined by MTT assay. (c) SH-SY5Y neurons were treated with Mog (0, 6.25, 12.5, and 25 *μ*M) for 2 h followed by costimulation with LPS (0.5 *μ*g/mL) for 18 h and the cell viability was determined by MTT assay. (d) BV-2 cells were treated with Mog (0, 6.25, 12.5, and 25 *μ*M) for 2 h followed by treatment with LPS (0.5 *μ*g/mL) for 18 h. Then, SH-SY5Y neurons were treated with the BV-2 cells-conditioned media for a further 24 h. The cell viability of SH-SY5Y neurons was examined by MTT assay. Similar results were obtained from three independent experiments. The values presented are the means ± SEM. ^*∗*^*p* < 0.05 and ^*∗∗*^*p* < 0.01 versus control group.

LPS, an endotoxin, can lead to neurotoxicity and neuroinflammation. Therefore, to explore whether Mog had a potential neuroprotective effect on BV-2 cells, we used LPS to induce neurotoxicity in BV-2 cells. Then, the cell viability was assessed by MTT assay. As shown in Figures 2(a) and 2(b), BV-2 cells were exposed to LPS for 24 h (0, 0.125, 0.25, 0.5, 1, and 2 *μ*g/mL) or treated with Mog for 24 h at different concentrations (0, 3.125, 6.25, 12.5, 25, and 50 *μ*M). However, the number of cells was significantly decreased after 2 *μ*g/mL of LPS treatment (Figure 2(a)). Treatment with Mog alone showed that the concentrations of Mog used did not induce any cytotoxic effect toward the viability of BV-2 cells (Figure 2(b)). Additionally, our findings revealed the conditioned media from BV-2 cells, which were treated with 100 *μ*L different concentrations of Mog (0, 6.25, 12.5, and 25 *μ*M) for 2 h, followed by stimulation with 0.5 *μ*g/mL of LPS for 18 h. Then, the conditioned media from the BV-2 cells cultures were treated with SH-SY5Y neurons for another 24 h, which can result in a significant decline in the number of SH-SY5Y neurons (Figure 2(d)) compared with directly stimulating SH-SY5Y neurons under the same conditions (Figure 2(c)). As shown in Figure 2(d), although Mog did not show any significant therapeutic effect, the cell viability of SH-SY5Y neurons is increasing as the concentration increases. Hence, we choose this concentration range for subsequent experiment.

### 3.2. Suppressive Effects of Mog on Proinflammatory Cytokine Responses in BV-2 Cells

**Figure 3:**
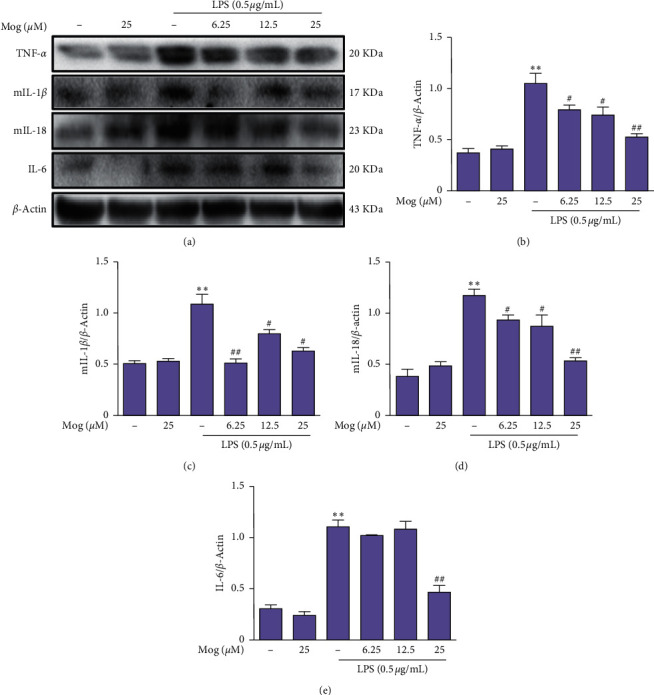
Suppressive effects of Mog on proinflammatory cytokine responses in BV-2 cells. (a) BV-2 cells were treated with Mog (0, 6.25, 12.5, and 25 *μ*M) for 2 h followed by the treatment with LPS (0.5 *μ*g/mL) for additional 18 h. The proteins expressions of TNF-*α*, mIL-1*β*, mIL-18, and IL-6 in six groups are determined by western blot. (b–e) Quantification of relative protein expression was performed by densitometric analysis. Similar results were obtained from three independent experiments. The values presented are the means ± SEM. ^*∗*^*p* < 0.05 and ^*∗∗*^*p* < 0.01 versus control group; ^#^*p* < 0.05 and ^##^*p* < 0.01 versus LPS group.

Under pathological conditions, proinflammatory cytokines are an important parameter of neurotoxicity and neuroinflammation. Our further work investigated the effect of proinflammatory cytokines in LPS-treated BV-2 cells and the Mog weather inhibited the generation of proinflammatory cytokines. At first, we added Mog (0, 6.25, 12.5, and 25 *μ*M) for 2 h. Then, stimulation with 0.5 *μ*g/mL of LPS was done for 18 h. As presented in Figure 3(a), we used western blot to assess the protein expressions of TNF-*α*, IL-1*β*, IL-18, and IL-6; and the results showed that Mog (25 *μ*M) dramatically decreased the protein expressions of inflammatory mediators, which were induced by LPS (Figures 3(b)–3(e)). Consistently, we consider that Mog exerted an antineuroinflammatory effect in the BV-2 cells activated by LPS.

### 3.3. Mog Attenuates LPS-Induced iNOS, COX-2, and HMGB1 Proteins Expression Increased in BV-2 Cells

**Figure 4:**
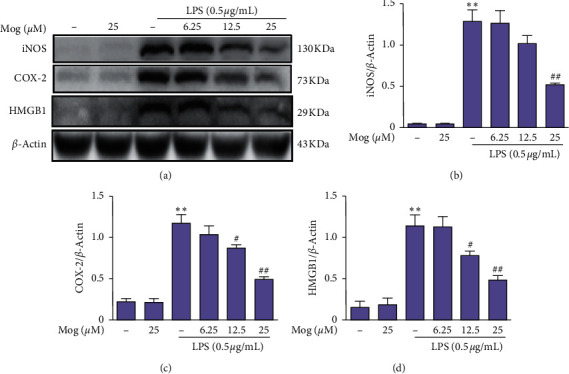
Mog attenuates LPS-induced iNOS, COX-2, and HMGB1 proteins expression in BV-2 cells. (a) BV-2 cells were pretreated with Mog (0, 6.25, 12.5, and 25 *μ*M) for 2 h followed by stimulation with LPS (0.5 *μ*g/mL) for additional 18 h. The expression levels of iNOS, COX-2, and HMGB1 were analyzed by western blot. (b–d) Quantification of relative protein expression was performed by densitometric analysis. Similar results were obtained from three independent experiments. The values presented are the means ± SEM. ^*∗*^*p* < 0.05 and ^*∗∗*^*p* < 0.01 versus control group; ^#^*p* < 0.05 and ^##^*p* < 0.01 versus LPS group.

To determine the effect of Mog on the expression of iNOS, COX-2, and HMGB1 proteins, BV-2 cells were treated with Mog (0, 6.25, 12.5, and 25 *μ*M) for 2 h, followed by treatment with or without LPS (0.5 *μ*g/mL) for 18 h. The data of western blot showed that the expressions of iNOS, COX-2, and HMGB1 were significantly increased by LPS treatment; however, these phenomena were reversed by 25 *μ*M of Mog treatment and demonstrated a dose-dependent reduction (Figure 4). These results suggested that Mog could correct the neuroinflammatory response induced by LPS.

### 3.4. Mog Suppressed the TLR4-Mediated NF-*κ*B and MAPKs Signaling Pathways in LPS-Treated BV-2 Cells

**Figure 5:**
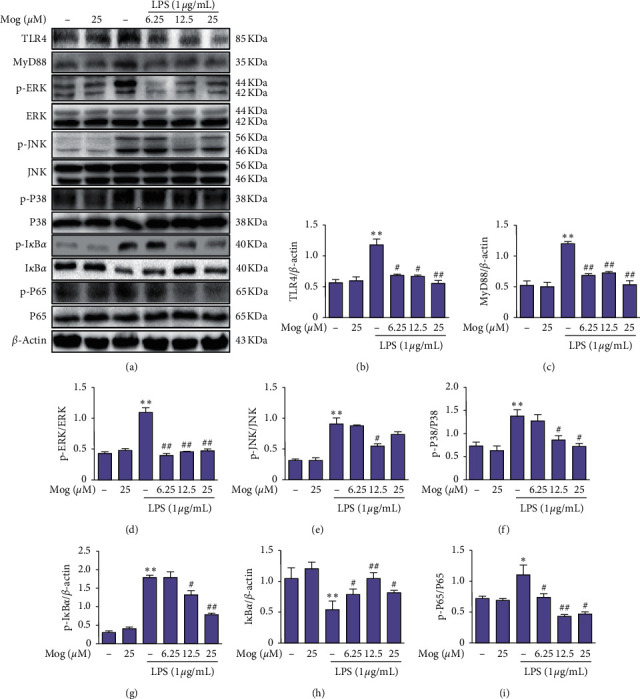
Mog suppressed the TLR4-mediated NF-*κ*B and MAPKs signaling pathways in LPS-treated BV-2 cells. (a) Cells were cultured with Mog (0, 6.25, 12.5, and 25 *μ*M) for 2 h and then treated with LPS (1 *μ*g/mL) for another 1 h. The proteins expressions of TLR4, MyD88, p-ERK, ERK, p-JNK, JNK, p-P38, P38, p-I*κ*B*α*, I*κ*B*α*, p-P65, and P65 were measured by western blot analysis. (b–i) Quantification of relative protein expression was performed by densitometric analysis. Similar results were obtained from three independent experiments. The values presented are the means ± SEM. ^*∗*^*p* < 0.05 and ^*∗∗*^*p* < 0.01 versus control group; ^#^*p* < 0.05 and ^##^*p* < 0.01 versus LPS group.

Considering that the TLR4/MyD88 signaling pathway as a proinflammatory signaling pathway plays a key role in modulating the expression of many proinflammatory proteins, we examined whether Mog could reduce TLR4/MyD88 signaling pathway. BV-2 cells were treated with Mog (0, 6.25, 12.5, and 25 *μ*M) for 2 h, followed by treatment with or without LPS (1 *μ*g/mL) for 1 h. By analyzing the results of Mog on these signaling pathways in BV-2 cells, we found that Mog significantly inhibited LPS-activated TLR4, MyD88, phospho-NF-*κ*B P65 (p-P65), and phospho-MAPK p38 (p-P38) proteins expressions (Figures 5(a)–5(c), 5(f), and 5(i)). Additionally, further study indicated that Mog significantly suppressed LPS-induced phosphoinhibitory subunit of NF-*κ*B (p-I*κ*B*α*), phospho-ERK (p-ERK), and phospho-JNK (p-JNK) protein expression increased in BV-2 cells (Figures 5(a), 5(d), 5(e), 5(g), and 5(h)). Therefore, these results proved that Mog dramatically decreased LPS-stimulated neuroinflammatory protein expression increased by inhibiting TLR4-mediated NF-*κ*B and MAPKs signaling pathways in BV-2 cells.

### 3.5. Mog Activation of Nrf2 Signaling Pathway in LPS-Treated BV-2 Cells

**Figure 6:**
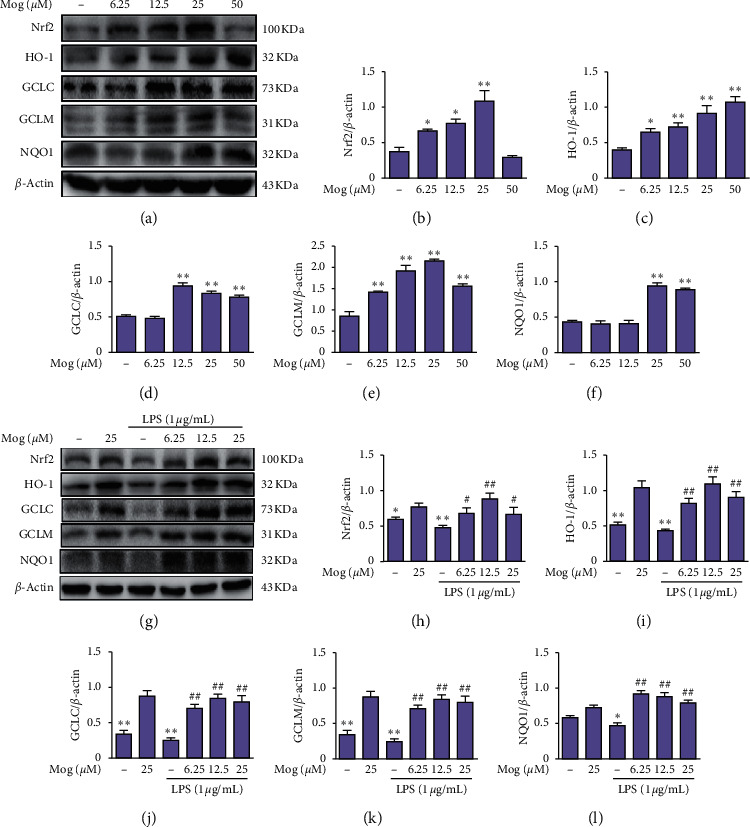
Mog activation of Nrf2 signaling pathway in LPS-treated BV-2 cells. (a) Different added concentrations of Mog (0, 6.25, 12.5, 25, and 50 *μ*M) in BV-2 cells for 2 h, and the proteins expressions of Nrf2, HO-1, GCLC, GCLM, and NQO1 were tested by western blot. (g) BV-2 cells were plated in 6-well plates, preincubated with Mog (0, 6.25, 12.5, and 25 *μ*M) for 2 h, and then challenged with or without LPS (1 *μ*g/mL) for 1 h. (b–f and h–l) Quantification of relative protein expression was performed by densitometric analysis. Similar results were obtained from three independent experiments. The values presented are the means ± SEM. ^*∗*^*p* < 0.05 and ^*∗∗*^*p* < 0.01 versus control group; ^#^*p* < 0.05 and ^##^*p* < 0.01 versus LPS group.

To further clarify the mechanism by which Mog reduces inflammation, we explored the potential involvement of Nrf2 and its downstream proteins, including HO-1, GCLC, GCLM, and NQO1. At first, BV-2 cells were treated with different concentrations (0, 6.25, 12.5, 25, and 50 *μ*M) of Mog for 2 h, and we found that Nrf2 and its downstream proteins expressions were dose-dependently increased, with the most remarkable change observed in the 25 *μ*M concentration (Figures 6(a)–6(f)). In addition, BV-2 cells were stimulated with 1 *μ*g/mL LPS for 1 h in the presence of Mog (0, 6.25, 12.5, and 25 *μ*M) for 2 h. The western blot analysis results revealed that LPS inhibited the expression of Nrf2 and its downstream proteins, while Mog changed this situation (Figures 6(g)–6(l)). These results indicate that Mog could also effectively blunt the LPS-induced inflammatory response of BV-2 cells.

### 3.6. Mog Promotes the AKT/AMPK Pathway Activation in BV-2 Cells

**Figure 7:**
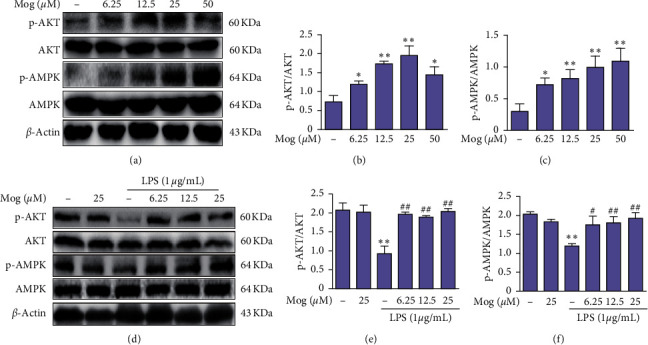
Mog can upregulate AKT/AMPK expression in BV-2 cells. (a) BV-2 cells were pretreated with Mog (0, 3.125, 6.25, 12.5, and 50 *μ*M) for 2 h, and whole cell lysates were subjected to western blotting analysis with AKT, p-AKT, AMPK, and p-AMPK. (d) BV-2 cells were treated with LPS (1 *μ*g/mL) for 1 h after incubation with various concentrations (0, 6.25, 12.5, and 25 *μ*M) of Mog for 2 h. Total protein extracts from different treatment for BV-2 cells were prepared for detecting AKT, p-AKT, AMPK, and p-AMPK. (b-c and e-f) Protein samples were analyzed by western blot with specific antibodies as described. Similar results were obtained from three independent experiments. The values presented are the means ± SEM. ^*∗*^*p* < 0.05 and ^*∗∗*^*p* < 0.01 versus control group; ^#^*p* < 0.05 and ^##^*p* < 0.01 versus LPS group.

Previous studies have indicated that the AKT/AMPK pathway is involved in inflammation. To further verify the anti-inflammatory mechanism of Mog, the AKT/AMPK signaling pathway is investigated after Mog (0, 6.25, 12.5, 25, and 50 *μ*M) treatment with BV-2 cells for 2 h. Western blotting showed that Mog promoted AKT and AMPK phosphorylation (Figures 7(a)–7(c)). Furthermore, BV-2 cells were treated with Mog (0, 6.25, 12.5, and 25 *μ*M) for 2 h, followed by exposure to LPS (1 *μ*g/mL) for 1 h, and western blotting was performed with anti-p-AKT and anti-p-AMPK antibodies. Results revealed that Mog increased LPS-inhibited p-AKT and p-AMPK levels in BV-2 cells (Figures 7(d)–7(f)). These data suggest that the AKT/AMPK pathway is essential for the ability of Mog to protect against LPS-inhibited p-AKT and p-AMPK expression.

### 3.7. AKT/AMPK-Nrf2 Pathway Mediated the Anti-Inflammatory Effect of Mog

**Figure 8:**
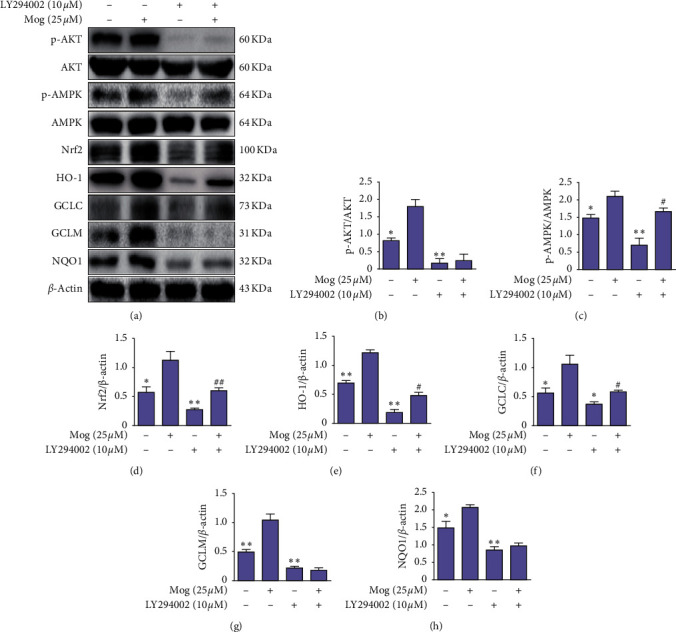
AKT/AMPK-Nrf2 pathway mediated the anti-inflammatory effect of Mog. (a) BV-2 cells were pretreated with LY294002 (10 *μ*M) for 18 h and then treated with Mog (25 *μ*M) for 3 h. The proteins expressions of AKT, p-AKT, AMPK, p-AMPK, Nrf2, HO-1, GCLC, GCLM, and NQO1 were analyzed by western blotting. (b–h) Protein samples were analyzed by western blot with specific antibodies as described. Similar results were obtained from three independent experiments. The values presented are the means ± SEM. ^*∗*^*p* < 0.05 and ^*∗∗*^*p* < 0.01 versus the Mog group; ^#^*p* < 0.05 and ^##^*p* < 0.01 versus the LY294002 group.

To further determine the upstream signaling pathway involved in Mog-mediated Nrf2 activation, we investigated the effects of LY294002, a specific inhibitor of AKT, on Nrf2 translocation. The BV-2 cells were treated with LY294002 (10 *μ*M) for 18 h, and then Mog was added (25 *μ*M) for 3 h. As shown in Figure 8, LY294002 dramatically inhibited AKT and AMPK phosphorylation and Nrf2 and its downstream proteins expression, and Mog can reverse this phenomenon, which suggested that Mog modulated Nrf2 translocation via the activation of the AKT/AMPK-Nrf2 signaling pathway in BV-2 cells.

## 4. Discussion

This is the first study to report that Mog exerts antineuroinflammatory effects in vitro by inhibiting microglial activation and the release of inflammatory mediators and proinflammatory cytokines and attenuating neuronal damage in LPS-induced neuroinflammation. Previous studies demonstrated that inhibiting inflammation is the key mechanism for the prevention of brain damage during neurodegenerative process, and activated microglia are an important criterion for the diagnosis of neurodegenerative diseases [24], because microglia are crucial immune cells in nervous system and interact with other brain cells by releasing inflammatory or proinflammatory factors and are widely researched on neuroinflammation [25]. On the other hand, microglia protected neuroinflammation function by removing protein aggregates, phagocytosing debris, and aiding repair [2]. In the present study, it was proved that several anti-inflammatory agents can inhibit microglial activation and thus attenuate neuroinflammatory effects and impede the development of related diseases [26].

Mog is the major bioactive constituent of Monk fruit, and Monk fruit is derived from the water extraction of the fruit *Siraitia grosvenorii* that mainly grows in Guangxi province in southern China [16]. In food industry, Mog was traditionally used as a sweetener [27]. From ancient times, *Siraitia grosvenorii* has been applied in Chinese Medicine treatment of lung congestion, the removal of phlegm, sore throat, and constipation in China [18]; and a lot of evidence showed that Mog has the same pesticide effects, such as anti-inflammation, anti-oxidative, and anticancer effects [18, 19, 28]. For example, Li et al.'s experimental results show that Mog through AMPK activation which regulates SREBP-1, PPAR-c, and PPAR-*α* and thus improves the imbalance between lipid acquisition and lipid removal improved physiological, therefore, improving the biochemical and pathohistological changes, it is proved that Mog has certain medicinal value [17]. In the present study, we investigated the effect of Mog on LPS-induced neuroinflammation in microglia. Our results revealed that Mog significantly reduced proinflammatory proteins and relevant inflammation pathway expression. Several lines of evidence proved that microglia play a vital role in the brains of AD patients and are crucial in the pathogenesis of the disease, due to the fact that the activation of microglia promotes a series of released proinflammatory factors that extremely impact the process of pathogenic condition with AD [29]. Additionally, various lines of evidence exhibited that microglial activation occurs by TLRs. Studies confirmed that TLR4 is connected with numerous neurodegenerative disorders, and inhibited TLR4 by function blocking antibodies or siRNA knockdown can inhibit released proinflammatory factors, such as IL-6 and TNF-*α* [30]. As we all know, TLRs are a major immune factor of innate immunity, and, as one member, TLR4 is activated by LPS. In the last years, several lines of evidence suggested that the activation of TLR4 binds to the adapter protein MyD88 and activates NF-*κ*B [31]. It is well known that NF-*κ*B family plays a vital role in inflammation. The activation of NF-*κ*B not only promotes the phosphorylation of proteins called inhibitors of kappa-B (I*κ*Bs), such as I*κ*B*α* that is mediated gene transcription, but also promotes the release of various proinflammatory proteins, including COX-2, iNOS, TNF-*α*, IL-1*β*, and IL-6 [32, 33]. Hence, we adopted BV-2 cell line for in vitro study and LPS was used to activate BV-2 cells. Our experimental results verified the above conclusions and LPS-induced microglia activation, activating TLR4 signaling pathway and leading to the increase of MyD88, NF-*κ*B, and inflammatory proteins expression. However, pretreatment with Mog can reverse this result. Consequently, Mog is a potential effective inflammation inhibitor. Additionally, several lines of evidence indicate that activated microglia and secretion of proinflammatory factors connect with MAPKs. For example, ERK and JNK are mainly expressed in neurons and astrocytes, and p38 MAPK is mainly expressed in microglia [34]. Previous studies illustrated that the activation of microglia further acts on neurons and astrocytes by promoting secretion of proinflammatory factors [35]. Our findings also displayed that conditioned media from LPS-treated BV-2 cells significantly weakened the viability of SH-SY5Y cells, while the decline was reversed after the Mog treatment. However, the same concentration of LPS or Mog, as in the microglial supernatants, had no effect on the viability of SH-SY5Y cells. Therefore, we can draw a conclusion that Mog is effective on neuronal cells against neurotoxicity. Moreover, it is reported that the blood-brain barrier (BBB) is the barrier between plasma and brain cells formed by the capillary wall of the brain and glial cells and between plasma and cerebrospinal fluid (CSF) formed by the choroid plexus, and these barriers prevent certain substances (mostly harmful) from passing from the blood to brain tissue [36]. Mog is easily soluble in water, and it is supposed to have a poor ability to cross the BBB, but, due to its small molecular weight, it can enter the brain through the junction between endothelial cells and astrocytes; and hydrophilic small molecules such as sucrose cross the BBB via a paracellular pathway along the concentration gradient between endothelial cells. The protective effect of Mog on BBB and its mechanism need to be further studied.

It is obvious that Nrf2 pathway plays a vital role against oxidative stress and anti-inflammation. Upon exposure to oxidative stress and anti-inflammation, Nrf2 translocates into the nucleus and binds to cis-acting antioxidant-responsive elements (AREs) and further promotes the transcription of a broad range of cytoprotective genes, including HO-1, GCLM, GCLC, and NQO1. Neurodegenerative diseases such as Alzheimer's, Parkinson's, and Huntington's are characterized by oxidative stress and increased inflammation of neurons. Nrf2's ability to reduce oxidative stress and associated neuroinflammation makes it an attractive therapeutic target for the discovery of novel therapeutic agents for the prevention and treatment of Parkinson's disease and other neurodegenerative diseases [37]. This research revealed that Mog has an anti-inflammatory effect by activating Nrf2 signaling pathway, and this activation of Nrf2 is AMPK- or AKT-dependent in LPS-challenged inflammatory response [12]. Multiple studies suggested that AKT plays a role in various biological processes, for example, cell growth, proliferation, apoptosis, and protein synthesis [38]. As a heterotrimeric serine/threonine kinase, AMPK involves a variety of metabolic stresses, including oxidative stress, inflammation, and hypoxia [39]. Our results revealed that AKT and AMPK involved LPS-induced neuroinflammation and activated Nrf2 pathway. The activation of Nrf2 pathway further regulated the expression of downstream detoxification and cytoprotective genes, such as HO-1, GCLM, GCLC, and NQO1. Our study further indicated that Mog can activate Akt and AMPK, thus increasing the expression of Nrf2 and its downstream proteins. Furthermore, Mog exerted a dose-dependent effect of increasing the expressions of AKT, AMPK, Nrf2, and downstream detoxification and cytoprotective genes.

**Figure 9:**
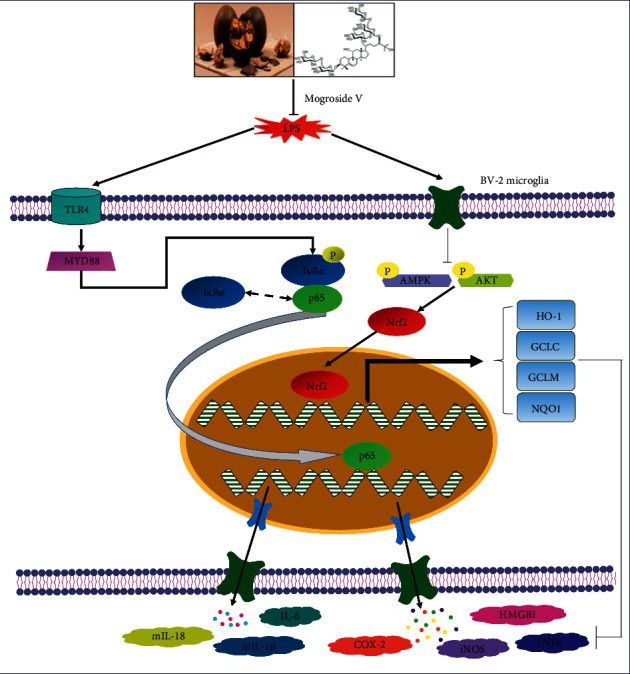
The scheme of mechanisms involved in the antineuroinflammatory effects of Mog in LPS-induced BV-2 cells.

In summary, as illustrated in Figure 9, the research and investigation on Mog proved that it plays an important role in neuroinflammation by inhibiting TLR4-MyD88 and activating AMPK/AKT-Nrf2 signaling pathways in LPS-stimulated BV-2 cells. Previous studies displayed that Mog has protective effects on LPS-induced ALI in mice; hence, our experiment indicates that Mog exhibits significant neuroprotective properties, so it is a promising target for the therapeutic approach for the treatment of neurodegenerative diseases.

## Data Availability

The datasets used and/or analyzed during the current study are available from the corresponding author upon reasonable request.
